# Contribution of Marital Distance to Community Inbreeding, Homozygosis, and Reproductive Wastage for Recessively Inherited Genetic Disorders in Madhya Pradesh, India

**DOI:** 10.4084/MJHID.2013.063

**Published:** 2013-11-04

**Authors:** R. S. Balgir

**Affiliations:** Department of Biochemistry, Regional Medical Research Centre for Tribals (ICMR), Madhya Pradesh, India.

## Abstract

**Background:**

Recessively inherited genetic disorders such as sickle cell anemia and β-thalassemia are commonly encountered in heterozygous and homozygous form in India. These hemolytic disorders cause a high degree of reproductive wastage in vulnerable communities. Inbreeding is usually the mating between two related individuals. Homozygosis is antagonistic process of heterosis.

**Purpose:**

This study was aimed at finding reproductive outcome in carrier couples of sickle cell anemia, and β-thalassemia in terms of reproductive wastage in relation to varied marital distance between partners in Madhya Pradesh.

**Methods:**

A total of 107 carrier couples, 35 and 72, respectively of β-thalassemia major and sickle cell anemia with confirmed affected offspring after taking detailed reproductive history were studied following the standard methodology in a tertiary hospital in Central India during March 2010 to February 2013.

**Results:**

A majority of sickle cell and β-thalassemia carrier couples, 77.8% and 65.7%, respectively, had married within physical distance of radius less than 50 kms. away from their native places. It was found that as the marital distance between two carrier partners of above disorders decreases, the number of abortions, still-births, neonatal mortality, infant mortality, and mortality under 10 years age increases, and vice versa, implicating inbreeding and homozygosis. The overall reproductive wastage of 28.2% and 18.6% was recorded in carrier couples of sickle cell disease and β-thalassemia, respectively. This combined reproductive wastage is negatively correlated (r= −0.74; p<0.001) to physical marital distance between the life partners.

**Conclusions:**

Relative small population size clubbed with small marital distance leads to inbreeding resulting in homozygosity which increases chances of affected offspring by recessive or deleterious traits and contributes to decreased fitness of a couple or population in Central India.

## Introduction

In a broad sense, it is necessary to consider that inbreeding in a population can take place under two quite different biological situations. There may be inbreeding because of restriction of population number. The degree of relationship between the individuals in a population depends on the size of that population since the individuals are more closely related to each other in a small population than in a large one. Thus, inbreeding, in general, is a phenomenon frequently associated with small populations. On the other hand, inbreeding can occur in a large population as a form of nonrandom mating when the frequency of consanguineous matings is higher than that expected by chance. In this case, the population shows an excess of homozygotes with respect to a random mating population in which genotypic frequencies are expected to be in Hardy-Weinberg equilibrium.^1^

Although a high proportion of marriages in Asia especially in India are consanguineous (i.e. contracted between close biological relatives), there is, in general, a dearth of demographic attributes, marital distance, and association between consanguinity and recessively inherited genetic disorders leading to homozygosis among tribal and nontribal communities in India. It would be interesting to study the effect of consanguinity on fertility, reproductive loss, and development disorders among tribal and nontribal communities in Central India. Consanguineous marriages may be classified as between uncle and niece, between first cousins, between first cousins once removed, between second cousins, between second cousins once removed, and between third cousins.^2^ It is presumed that the frequency of developmental anomalies, in general, is significantly more among the children of consanguineous parents.

The present study is designed with the aims and objectives of finding the reproductive outcome in carrier couples of sickle cell anemia, and β-thalassemia in terms of abortions, still-births, neonatal mortality, infant mortality, and mortality less than 10 years of age, in relation to marital distance in the population of Central India. This study illustrates and indirectly implicates the prevalence of consanguinity based on the matrimony and physical distance between the two mating partners in the population of Madhya Pradesh in Central India.

## Materials and Methods

A total of 107 carrier couples, having confirmed offspring of either β-thalassemia major (35) or sickle cell anemia (72) routinely referred by the experts (in Gynaecology, Pediatrics, and Blood Bank) to us for further investigations, who were attending Netaji Subhash Chandra Bose Medical College & Hospital at Jabalpur in Madhya Pradesh of Central India were included in the study during the period from March 2010 to February 2013. Subjects with other hemoglobinopathies, hematological disorders or other causes of anemia were excluded from the study. Ethical clearance was obtained from Human Ethical Committee of RMRCT (ICMR) Jabalpur. Detailed reproductive history of each couple was recorded like total number of conceptions, abortions, miscarriages or still-births, live-births, surviving children, infant or neonatal deaths, marital distance between the two partners (native place), etc.

Intravenous 2–3ml blood was taken under aseptical conditions from each individual after taking informed/written consent for screening of sickle cell anemia and β-thalassemia syndrome. Laboratory investigations were carried out following the standard procedures after cross checking for quality control from time to time. Hematological parameters were studied by using an automated Blood Cell Counter (Model-MS_5_9, Melet Schloesing Laboratories, Cergy-Pontoise Cedex, France).

The sickling test was performed using 2% freshly prepared sodium metabisulphite solution as reducing agent for the absence or presence of sickle cell hemoglobin.^3^ The routine hemoglobin lysate electrophoresis was carried out on cellulose acetate membrane (CAM) in Tris-EDTA-Borate buffer at pH 8.9 and quantification of A_2_ fraction of adult hemoglobin was done by elution method.^3^,^4^ The value more than 3.5% of A_2_ fraction of adult hemoglobin was taken as cut off point for determining the β-thalassemia trait. Those individuals having very high hemoglobin A_2_ value, i.e. more than 10% were suspected to have A_2_ plus hemoglobin E; and the test was confirmed by the investigations of other family members. Estimation of fetal hemoglobin was done according to technique described by Weatherall.^4^ The diagnosis of sickle cell- β-thalassemia was based on the findings of hemoglobin A, F, S and A_2_ on electrophoresis under alkaline pH, elevated (>3.5%) A_2_ levels.^5^ All the doubtful cases were further subjected to hemoglobin variant analysis for detecting any discrepancy (made for Bio-Rad Diagnostics, Hercules California, USA). Results were given to parents for treatment and further clinical management by concerned referring doctor. All the carriers/affected persons were imparted genetic/marriage counseling.

## Statistical Analysis

For the present study, a software package of STATA 11 was used to calculate the coefficient of correlation (r) to know the relationship between the marital distance (in Km) and reproductive wastage (combined abortions, still-births, neonatal deaths, infant mortality).

## Results

It is interesting to note that the majority of the sickle cell carrier couples (77.8%; 56/72) had married within physical distance of radius less than 50 kms. of their native places and only 22.2% (16/72) couples married beyond the radius of 50 kms. It is apparent from Table 1 that as the physical distance (in kilometers) between the native places of the two carrier partners of sickle cell anemia decreases the number of abortions, still-births, neonatal mortality, infant mortality, and mortality less than 10 years of age also go on increasing, and vice versa. The number of abortions (7%) and mortality under 10 years (11%) in proportion to total number of conceptions is quite high in this population. The overall reproductive wastage of 28.2% was recorded in the carrier couples of sickle cell anemia in Central India.

The marital distance as a whole for sickle cell anemia is negatively highly correlated (r = −0.74; p<0.001) with reproductive wastage (combined abortions, still-births, neonatal deaths, infant mortality). The marital distance of less than 50 km radius has shown very strongly negative (inverse) correlation (r = −0.88; p<0.001) with reproductive wastage (all subgroups such as abortions, still-births, neonatal deaths, infant mortality, and deaths under 10 years), whereas, the marital distance of less than 50 km radius has shown a very strong negative correlation (r = −0.84; p<0.001) with reproductive wastage; i.e. combined abortions, still-births, neonatal deaths, and infant mortality.

About two-third (65.7%) of the carrier couples of β-thalassemia got married within the radius of less than 50 kms. away from their native place, whereas, 34.3% (12/35) of them married beyond the radius of 50 kms. Table 2 shows the similar trend in carrier couples of β-thalassemia for marital distance as stated above for the sickle cell anemia. The number of abortions (4.6%) and mortality under 10 years (7%) in proportion to total number of conceptions is quite high in carrier couples of β-thalassemia. The overall reproductive wastage of 18.6% was recorded in the carrier couples of β-thalassemia in Central India.

However, the picture of marital distance for β-thalassemia with respect to correlation of reproductive wastage is not clear from Table 2 although similar trends as that of sickle cell anemia are anticipated. It may be relevant to mention here that β-thalassemia is not as common as sickle cell anemia in central India. Therefore, comparatively only half the number of sickle cell anemia carrier couples (72) were available for β-thalassemia (35) in the present study.

## Discussion

It is surprising that as the physical marital distance between the two carrier partners of sickle cell anemia or β-thalassemia major decreases the number of abortions, still-births, neonatal mortality, infant mortality, and mortality under 10 years of age go on increasing in the population of Central India as supported by the coefficient of correlation (r = −0.74; p<0.001). This is because of the fact that the mating of parents who are genetically related to each other and leads to some degree of inbreeding resulting in increased reproductive wastage but the illiterate rural people in Madhya Pradesh state are not aware of this fact. Inbreeding results in increased homozygosity, which can increase the chances of offspring being affected by recessive or deleterious traits. This generally leads to a decreased fitness of a couple or population.^6^ The inbreeding can also occur sometimes, when in a widely spread/scattered endogamous community in a larger area, prefer to marry within a short or limited physical distance for interactive closeness for convenience without knowing the fact that they are going to marry to a blood relative. It must be kept in mind that those villagers concentrated in particular surrounding area who have settled there in the distant past, are the ultimately blood relatives and belong to same original stock and share the common normal or abnormal gene pool of a community. Therefore, those communities who have small population size and marry randomly within physically small area by practicing community endogamy do have some degree of inbreeding that results in homozygosity of recessively inherited genetic disorders such as sickle cell anemia or β-thalassemia in the population under study, which is probably the case in Central India. The random marriage within a short physical marital distance is an additional dimension of inbreeding to the previously two situations described earlier[1]. In short, small population size clubbed with small marital distance leads to inbreeding resulting in homozygosity which increases chances of offspring being affected by recessive or deleterious traits and decreases fitness of a couple or population (r = −0.88; p<0.001).

The practical implications of the present study are many folds.^7^,^8^ Apart from the detection of homozygous sickle cell anemia or β-thalassemia major cases in the remote, rural, and backward population where the vulnerable people harbor the defective recessive genes in India, the major emphasis should be on avoiding the inbreeding in the community. The most appropriate approach would be imposition of prohibitions on marriages among blood relatives. In a situation where it is difficult to ascertain the common descent, the marriages should take place in the community as far off places (more than the radius of 50 kms. away) as possible. The outcome of homozygosity is burdensome, deleterious, pathetic, and traumatic to the patient as well as to the carrier couples.^9^,^10^ Therefore, prevention is better than cure.

The poverty stricken people who are the genetic victims of these recessively inherited disorders such as sickle cell anemia or β-thalassemia major, have to bear the brunt, therefore, exhaustive screening campaign should be launched by providing both necessary infrastructural facilities of neonatal and prenatal screening as well as technological knowhow at Primary Health Centre (PHC) level after adequately imparting training to the paramedical staff and the attending doctor for proper clinical management of the affected persons. The volunteer organizations such as NonGovernmental Organizations (NGOs) should be made responsible for spreading the message of bringing awareness among the at risk communities for carrier detection.^11^ The message should be clear that a carrier of sickle cell anemia or β-thalassemia major should not marry a carrier. Avoid marriage among close blood relatives. Strengthening of economical/financial support to these dwindling masses should be over emphasized in this preventive campaign. Let us hope that this approach will not only be successful but bear fruits also in the long run. India will certainly shine with healthy future generations!

## Conclusion

It is not only the small population size or consanguineous mating (among blood relatives) of the parents that results in inbreeding or increased homozygosity and, consequently, leads to lower fitness of the offspring or as such of the population. Contrary to this notion, when the marital distance is small between two mating parents, although random mating takes place practicing community endogamy, that also leads to some degree of inbreeding and increases homozygosity of recessively inherited genetic disorders such as sickle cell anemia or β-thalassemia affecting reproductive outcome in the vulnerable couples or population especially in Central India.

## Figures and Tables

**Table 1:** Reproductive wastage in carrier couples of sickle cell disease in relation to their marital distance in Madhya Pradesh, India.

Marital Distance (Km)	No. of Couples (N)	Total No. of Conceptions	No. of Abortions		No. of Stillbirths		Neonatal Mortality•		Infant Mortality▪		Mortality below 10 years	
			n	%	n	%	n	%	n	%	n	%
<10	14	46	7	15.2	3	6.5	3	6.5	5	10.9	5	10.9

11–20	12	42	4	9.5	0	0.0	0	0.0	1	2.4	9	21.4

21–30	12	36	2	5.5	0	0.0	1	2.8	1	2.8	1	2.8

31–40	10	28	0	0.0	1	3.6	2	7.1	2	7.1	4	14.3

41–50	8	22	0	0.0	0	0.0	0	0.0	0	0.0	3	13.6

51–60	2	11	0	0.0	0	0.0	0	0.0	0	0.0	0	0.0

61–70	3	11	2	18.2	1	9.1	0	0.0	0	0.0	0	0.0

71–80	1	3	0	0.0	0	0.0	1	33.3	0	0.0	1	33.3

81–90	1	4	0	0.0	0	0.0	0	0.0	0	0.0	0	0.0

91–100	1	2	0	0.0	0	0.0	0	0.0	0	0.0	0	0.0

101+	8	22	1	4.5	2	9.1	0	0.0	0	0.0	2	9.1

Total	72	227	16	7.0	7	3.1	7	3.1	9	4.0	25	11.0

• Birth to 28 days (Neonatal Mortality).

▪ Birth to 365 days or within 1 year (Infant Mortality).

**Table 2:** Reproductive wastage in carrier couples of β-thalassemia major in relation to their marital distance in Madhya Pradesh, India.

Marital Distance (Km)	No. of Couples (N)	Total No. of Conceptions	No. of Abortions		No. of Stillbirths		Neonatal Mortality•		Infant Mortality▪		Mortality below 10 years	
			n	%	n	%	n	%	n	%	n	%
<10	12	27	2	7.4	1	3.7	1	3.7	1	3.7	1	3.7

11–20	5	11	0	0.0	0	0.0	0	0.0	0	0.0	1	9.1

21–30	1	5	0	0.0	0	0.0	0	0.0	0	0.0	0	0.0

31–40	2	4	0	0.0	0	0.0	0	0.0	0	0.0	0	0.0

41–50	3	9	0	0.0	0	0.0	1	11.1	1	11.1	1	11.1

51–60	-	-	-		-		-		-		-	-

61–70	1	2	0	0.0	0	0.0	0	0.0	0	0.0	0	0.0

71–80	1	2	0	0.0	0	0.0	0	0.0	0	0.0	0	0.0

81–90	-	-	-		-		-		-		-	-

91–100	3	9	0	0.0	0	0.0	0	0.0	1	11.1	2	22.2

101+	7	17	2	11.8	0	0.0	0	0.0	0	0.0	1	5.9

Total	35	86	4	4.6	1	1.2	2	2.3	3	3.5	6	7.0

• Birth to 28 days (Neonatal Mortality).

▪ Birth to 365 days or within 1 year (Infant Mortality).
